# Beyond the *BRCA1/2* genes in ovarian cancer: the role of germline pathogenic variants in the *ATM* gene

**DOI:** 10.1007/s11033-025-10357-x

**Published:** 2025-02-21

**Authors:** Daniele Guadagnolo, Angelo Minucci, Antonella Chiavassa, Gabriella Gentile, Francesca Salvatori, Nader Khaleghi Hashemian, Giulia Maneri, Maria Piane, Simona Grotta, Paola Grammatico, Antonio Pizzuti, Daniele Santini, Laura De Marchis

**Affiliations:** 1 Department of Experimental Medicine, School of Medicine and Dentistry, Sapienza University of Rome, 00185 Rome, Italy; 2 Departmental Unit of Molecular and Genomic Diagnostics, Fondazione Policlinico Gemelli IRCCS, Rome, Italy; 3 Department of Radiological, Oncological and Pathological Science, School of Medicine and Dentistry, Sapienza University of Rome, 00161 Rome, Italy; 4 Sant ‘Andrea University Hospital, 00189 Rome, Italy; 5 Department of Clinical and Molecular Medicine, School of Medicine and Psychology, Sapienza University of Rome, 00189 Rome, Italy; 6 Laboratory of Medical Genetics, San Camillo-Forlanini Hospital, 00152 Rome, Italy; 7 Department of Medico-Surgical Sciences and Biotechnology, Polo Pontino, Sapienza University of Rome, Rome, Italy; 8 Oncology Unit, Department of Hematology, Oncology and Dermatology, Umberto I University Hospital, 00161 Rome, Italy

**Keywords:** *ATM*, Hereditary Breast and Ovarian Cancer, Ovarian cancer, Cancer prevention, Moderate penetrance genes, Risk-Reducing Bilateral Salpingo-oophorectomy

## Abstract

**Background:**

Ovarian Cancer (OC) prevention and early-stage detection represents a challenge due to the lack of effective surveillance. The identification of high-risk women is crucial as it provides access to prophylactic oophorectomy and reduces disease burden. Next-Generation Sequencing approaches enable the investigation of several genes associated with monogenic hereditary cancer predisposition, including ovarian cancer. For family members of patients affected by ovarian cancer without identification of a germline pathogenic variant, despite the increased empirical risk (3 times) of ovarian cancer incidence, prophylactic surgery is not indicated but may be suggested as the only efficient strategy.

**Methods and Results:**

We hereby present 2 cases of OC in which a germline heterozygous pathogenic variant in the ATM gene was identified: the first in the contest of Hereditary Breast and Ovarian Cancer (HBOC) family history and, in the other one, a late onset of neoplasms, to underline the importance of defining guidelines and management of moderate penetrance genes variants also for ovarian cancer prevention.

**Conclusions:**

Carriers of heterozygous pathogenic variants in the *ATM* gene have an increased risk of neoplasms incidence, mostly breast but also of OC with an absolute estimated risk of 2–3 times greater than the general population. For these patients there is not well-established evidence of benefit in risk reducing bilateral Salpingo-oophorectomy.

## Introduction

Ovarian cancer (OC) occupies the eleventh place for incidence among female cancers in western populations and has a significant health burden due to the late and non-specific symptoms and the absence of validated screening strategies that could allow early diagnosis. A woman’s life-time risk for ovarian cancer is about 1/70, 1.3% [1] 

Despite the important improvements in the treatment in the last decade, ovarian cancer remains the most lethal gynecologic malignancy [2].

A significant portion of cases of OC, accounting for more than 20%, appears to be associated with monogenic cancer susceptibility conditions, of which the majority (up to 90%) is due to heterozygous pathogenic or likely pathogenic (P/LP) variants in the *BRCA1/2 (BRCA)* genes [1, 3]. However, PVs in other genes involved in DNA-damage repair pathways, especially in Homologous Recombination Repair (HRR), are associated with Hereditary Breast and Ovarian Cancer (HBOC) [4]. These genes are classified as high-risk or moderate-risk genes based on the increase of breast and/or tubo-ovarian cancer risk. A fourfold increase in risk defines high penetrance genes, such as *BRCA1/2* [5].

In this scenario the *ATM* gene (Ataxia-Telangiectasia Mutated, MIM *607585) is one of the master regulators of Homologous Recombination (HR) DNA repair in response to Double-Strand Breaks (DSBs) and is one of the possible monogenic causes of hereditary cancer susceptibility [6]. Homozygous or compound heterozygous pathogenic variants in *ATM* gene are associated with Ataxia-Telangiectasia (MIM #08900), a multisystemic disorder with neurological and immunological involvement, and marked susceptibility to hematological and solid malignancies with autosomal recessive inheritance, including OC [7, 8]. It is classified as a moderate-penetrance gene [9, 10] has been demonstrated that high ATM protein tumor expression correlates with adverse prognosis in ovarian cancers [11], while ATM deficiency appears to be associated with better prognosis in several cancer types, especially after Poly ADP-Ribose polymerase inhibitors (PARPi) treatment [12]. For these reasons, specific ATM-inhibitor drugs to enhance PARPi response are under investigation [13].

Hereditary cancer syndromes are a prime target for early detection and prevention. The investigation of the possible causes of HBOC is therefore of key importance to improve current treatment strategies in several cases and concurrently identify candidate families for genetic testing and tailored surveillance [14]. Germ-line genetic testing for a panel of cancer-susceptibility genes is recommended in all epithelial ovarian cancer cases [13], but it is not always available in clinical practice, and, in some cases, it is limited to *BRCA1*/*2*. Moreover, somatic testing for Homologous Recombination Deficiency (HRD) is recommended in advanced high-grade ovarian cancers [2, 15, 16].

The standard of care for women with *BRCA1* and *BRCA2* P/LP variants is risk-reducing bilateral surgical resection of the fallopian tubes and ovaries (RRBSO) [2, 15]. Nevertheless, there is still no consensus on the management of OC risk in individuals and families harboring a heterozygous *ATM* pathogenic variant. OC screening in *ATM* carriers is not contemplated in the current NCCN guidelines, and available evidence is insufficient to establish benefit from RRBSO [15].

We report two cases of ovarian cancer in individuals with germline pathogenic *ATM* variants, discussing the therapeutic strategies adopted. The aim of this paper is to highlight the need for a better definition of the cumulative risk for these patients, a crucial step towards the definition of a more appropriate surveillance path.

## Results

### Case A

A 59-year-old woman (A; III:12, Fig. 1), in good clinical conditions and no comorbidities was referred in March 2022 for hereditary cancer genetic counseling.

**Fig. 1:**
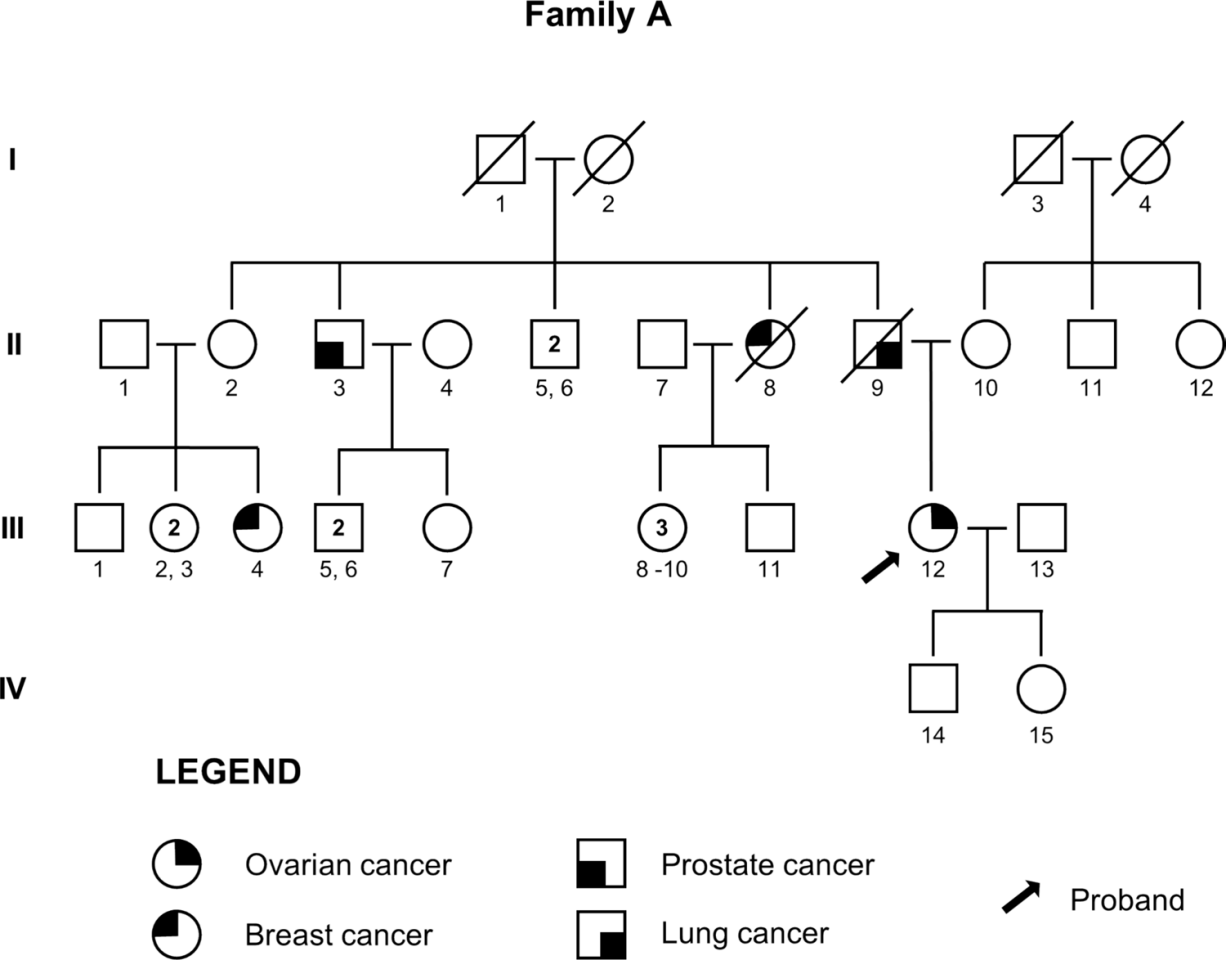
Pedigree of the family of Case A

The patient had a family and personal history of oncological disease. Her father (A; II:9) died of lung cancer at the age of 78, a paternal uncle due to prostate adenocarcinoma at the age of 82 (A; II:3), and a paternal aunt died of breast cancer at 42 years of age (A; II:8). She also reported a deceased female cousin on the paternal side (A; III:4) who had breast cancer at the age of 33 years (A; III:4).

The patient received a diagnosis of bilateral borderline mucinous ovarian tumor and underwent hysterectomy and bilateral salpingo-oophorectomy, with peritoneal biopsies and peritoneal washing in November 2012 (age 49).

Tumor markers were negative at the time of surgery.

From January to June 2013, she received adjuvant therapy with Carboplatin and Paclitaxel every 3 weeks for 6 cycles, with good tolerance [2].

In April 2014 a control CT scan revealed a modest dimension increase in a previously known hypovascular oval lesion in the peritoneum. A subsequent PET/CT scan, performed in May 2014, did not show increased metabolic activity suggestive for active disease.

In June 2014 an increase in the serum tumor marker CA-125 value was noted (28.88 U/ml. Normal values < 30 U/ml) and the patient underwent a diagnostic and operative laparoscopy with appendectomy and biopsies with negative histological results.

In March 2016 CT scan showed a periumbilical formation with a 6 cm diameter, which was confirmed to be metabolically active by PET/CT scan.

In May 2016 the patient underwent surgical removal of the abdominal lesion and was diagnosed with a low-grade (G1) papillary serous carcinoma.

Until October 2016 the patient received 6 cycles of Carboplatin and Taxol. From cycle V Carboplatin was replaced by Cisplatin due to allergic reaction. Follow-up controls were negative until April 2021, when pelvis MRI revealed the presence of three formations in the left pelvis and one in the right iliac region, compatible with disease recurrence. Serum tumor markers increased, with a CA-125 value of 73 U/ml.

Transverse colon resection, associated with the removal of a lesion in the right iliac fossa and right pelvic lymphadenectomy, was performed in May 2021 with a diagnosis of High-Grade Serous Ovarian Cancer (HGSOC) [1]. A pathological review of the specimens for the previous neoplasms was proposed to the patient, but the samples were not available.

From June to December 2021 the patient underwent 7 cycles of Caelyx 20 mg/m^2^ and Trabectedin 0.9 mg/m^2^ every three weeks [2].

At initial evaluation, due to the familial and personal oncological history, molecular testing for the *BRCA* genes was proposed and undertaken but yielded negative results. Given the high suspicion, genetic testing for a panel of cancer susceptibility genes (*ATM, PALB2, CDH1, CHEK2, NBN, PTEN, STK11, TP53, BRIP1, MSH2, MLH1, MSH6, PMS2, EPCAM, RAD51C, RAD51D, APC*) was subsequently proposed. Next Generation Sequencing (NGS) analysis revealed the germline NM_000051.4: c.6450dup variant in the *ATM* gene in heterozygosity, a frameshift variant with a premature stop codon (10 codons downstream). The stop occurs before the most distal known pathogenic C-terminal truncations in the last exon. It is rare, as it is not reported in the GnomAD population database (v4.1 all samples and v3.1.1 non-cancer dateset). It is not reported in the scientific literature. It is reported in the ClinVar database (Variation ID 1076920) as pathogenic (two entries). The variant can be classified as pathogenic according to the American College of Medical Genetics and Genomics/Association for Molecular Pathology (ACMG/AMP) criteria as specified by the ATMspecificClinGenHereditaryBreast,OvarianandPancreaticCancer(HBOP)VariantCurationExpertPanel(VCEP) (https://cspec.genome.network/cspec/ui/svi/doc/GN020?version=1.3.0, Released Date 3/27/2024) with the PVS1, PM2_supporting, and PM5_supporting criteria. The PVS1 criterion can be applied at full strength according to the most recent recommendations, as frameshift variants occurring in that region of the gene are predicted to undergo Nonsense-Mediated Decay [16–20]. First-degree relatives were not available for genetic counseling to propose cascade testing.

### Case B

An 81-year-old woman (B; III:6, Fig. 2), in good clinical condition, was evaluated in May 2021. We collected familial oncological history and oncological patient anamnesis.

**Fig. 2:**
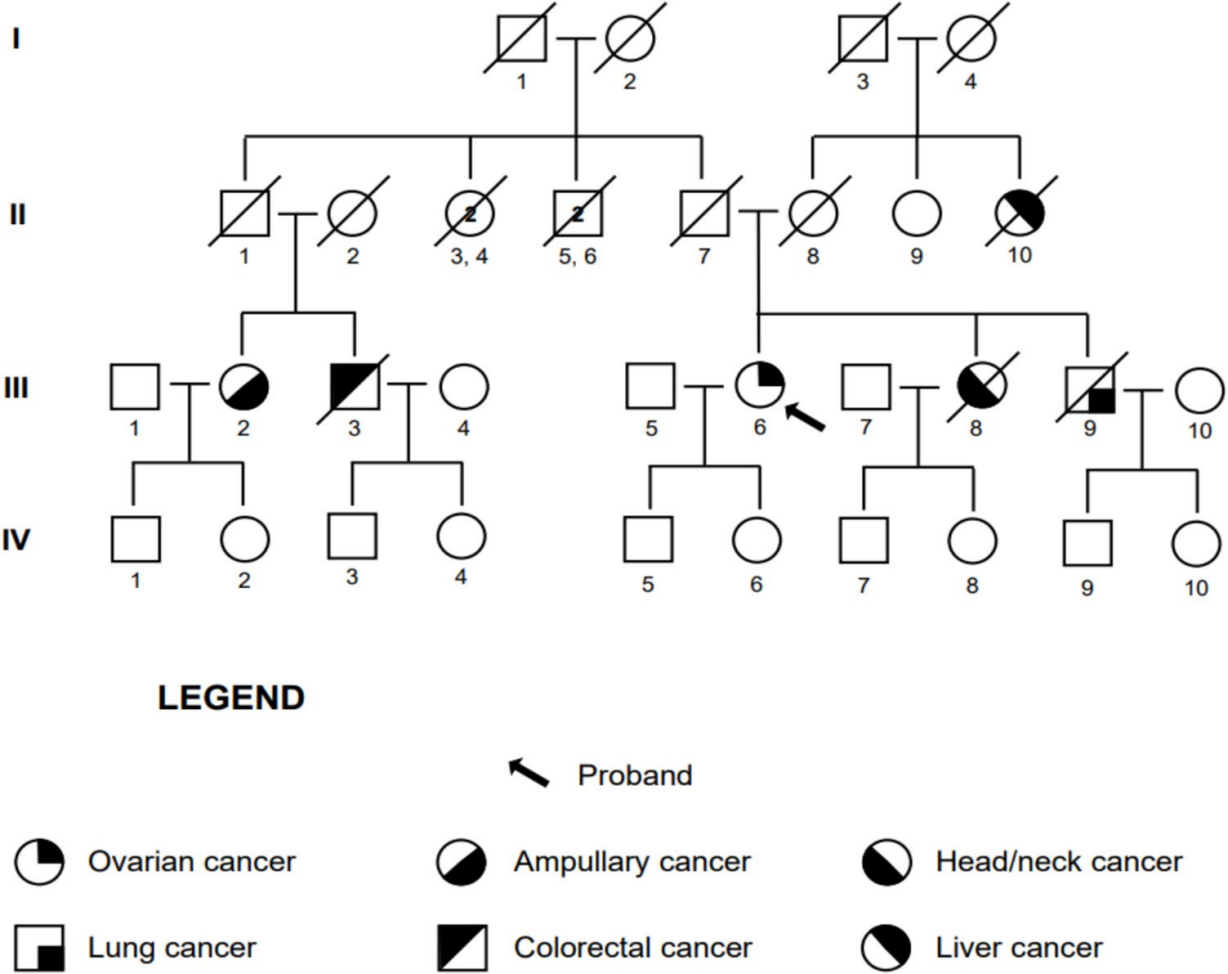
Pedigree of the family of Case B

The patient had a family history of oncological disease. Of her two siblings, her sister (B; III:8) died with head/neck cancer at 60 years of age, while her brother (B; III:9) died with lung cancer at age 76. A maternal aunt (B; II:10) died with an unspecified liver neoplasm. She also reported a paternal female cousin (B; III:2) diagnosed with ampullary cancer at age 60, and a male paternal cousin (B; III:3) deceased due to colorectal cancer.

In August 2020 the patient was admitted to the hospital for abdominal pain. Lower abdominal CT scan identified left an adnexal expansive formation (36 × 20 mm) with multiple peritoneal implants and a bilateral globular adenopathy in the obturator iliac chain. A subsequent lower abdomen MRI confirmed the extension of the implants in the Douglas excavation associated with peritoneal implants.

At serum tumor markers assessment, CA-125 was increased with a value of 77 U/ml (normal values < 30 U/ml), while others were within normal ranges.

Exploratory laparoscopy, performed in September 2020, identified a left ovarian mass involving the sigma and pelvic peritoneum, suggesting a peritoneal carcinosis. The lesions were sampled. Histological analysis allowed the diagnosis of HGSOC.

From October 2020 to January 2021, due to age and patient’s comorbidities,4 cycles of weekly Paclitaxel 80 mg/ m^2^ and Carboplatin AUC 2 were performed with good tolerance [2].

CT scan in February 2021 showed partial response with the persistence of peritoneal carcinosis nodules.

In March 2021 total hysterectomy with removal of pelvic peritoneum and bilateral adnexectomy with radical omentectomy was performed [GB7].

A genetic test for *BRCA1* and *BRCA2* genes in April 2021 was negative.

From April 2021 to May 2021 other 2 cycles of weekly Paclitaxel and Carboplatin were performed with good tolerance. PET/TC in July 2021 confirmed a complete radiological response.

In July 2021, the patient underwent hereditary cancer genetic counseling. Molecular testing for a panel of cancer susceptibility genes was undertaken (*ATM, BRIP1, CHEK2, DICER1, MLH1, MSH1, MSH6, PMS2, RAD51C, RAD51D*). NGS analysis identified the NM_000051.4: c.5347_5350del, p. (Glu1783ThrfsTer9) germline variant in heterozygosity in the *ATM* gene. This frameshift variant is predicted to cause a premature termination codon in a biologically relevant exon leading to nonsense mediated decay. It is very rare, being reported in 1/1612838 alleles in the GnomAD population database (v4.1). It is absent from the GnomAD v2.1.1 exome-only, non-cancer dataset. It is reported in the scientific literature as associated with Ataxia-Telangiectasia in compound heterozygosity with a different variant [21]. It is reported in the ClinVar database as pathogenic (Variation ID 1075452, four entries). The variant can be classified as pathogenic according to the ACMG/AMP criteria as specified by the *ATM*-specific ClinGen HBOP VCEP (https://cspec.genome.network/cspec/ui/svi/doc/GN020?version=1.3.0, Released Date 3/27/2024), with the PVS1, PM2_Supporting, PM5_supporting, PM3_supporting criteria. Genetic counseling for at-risk individuals of the family was proposed, and the son and daughter of the proband (B; IV:5; B; IV:6) and the four nephews (B; IV:7, B; IV:8, B; IV:9, B; IV:10) tested negative for the *ATM* PV.

Given the molecular findings, in June 2021, a maintenance treatment with Niraparib 200 mg was proposed. The treatment was discontinued in September 2021 due to myelotoxicity.

The following control CT scan in October 2021 confirmed the maintenance of radiological complete response with negative tumor markers. At present, the patient is undergoing clinical, biochemical, and instrumental follow-up, with no evidence of disease recurrence.

## Discussion

OC is the fifth leading cause of cancer-related mortality in women. Two-thirds of cases are diagnosed in advanced stages with an estimated 5-year survival rate of 20–40%, while a diagnosis in early stages raises this rate to about 90% [2, 12]. The GLOBOCAN study predicted a worldwide increase of 55% in OC incidence and 67% increase in mortality from years 2012 to 2035 [22]. If such inferences prove to be true, considerable efforts are still needed to intervene promptly in the management of this condition.

Although the role of familial history remains an important risk factor, genetic causes represent a significant proportion of cases and conversely, approximately half of individuals who harbor PVs in HBOC genes do not have a suggestive family history [5].

*ATM* is a moderate-penetrance cancer susceptibility gene and is one of the HR DNA regulators [6]. An association between pathogenic heterozygous *ATM* variants and cancer susceptibility was first noted for breast cancer (MIM #114480), when a moderately increased risk was demonstrated for carrier women, first in Ataxia-Telangiectasia families [7], then in the general population [23, 24]. Subsequently, a possible association with heterozygous *ATM* pathogenic variants was proposed for different malignancies, including OC [7, 8, 24–27]. The reported Odds Ratio for OC risk compared to the general population varies from 1.5 to 2.5 for most studies, resulting in a moderate-to-low risk increase [28–30]. The absolute risk for OC for *ATM* pathogenic variant female carriers appears to be 2–3% [15]. The association between *ATM* and specific OC histotypes has not been fully ascertained. As mentioned before, it has been also demonstrated that the tumor expression of ATM protein expression correlates with prognosis in ovarian cancers [9, 10].

Aside from diagnostic and familial implications, PVs or LP variants in genes associated with HRD can be assessed to guide clinical management towards prophylactic surgery in primary prevention and grant access to PARPi [15]. HRD as a potential therapeutic target has been initially investigated in *BRCA-*related ovarian neoplasms and its potential role in diagnosis and treatment has been demonstrated also in cases due to Loss-of-Function (LOF) of other genes implicated in DSB repair and HRR [12, 31].

The investigation of these other possible causes of HBOC is therefore of key importance [12]. The use of molecular testing to detect both high and moderate penetrance genes is fundamental and multi-gene NGS panels play a main role in this field, and they have proven to have a better cost-effectiveness compared to single-gene approaches in providing valuable clinical information [12, 32]. These tests are therefore recommended in all epithelial OC cases at diagnosis [15] and should be routinely applied to facilitate relevant decision-making for surgery, PARPi access and individual and familial risk management. Although BRCA1/2 pathogenic variants (PVs) are reliable predictors of sensitivity to PARPi in OC, current biomarkers for non-BRCA HRR PVs are inadequate to guide PARPi use. [33]

Despite the clear health impact of such analyses, in clinical practice there are still problems in their use. First, although the current international indications, clinical practice often differs across or within countries based on regional guidelines or test availability. Further difficulties arise for those patients whose initial care is dated before the systematic application of multigene testing. Furthermore, the absence of specific oncogenetics expertise in treatment-oriented teams might delay the molecular diagnosis for some patients. In the cases discussed in the present work, for example, the multigene evaluation was carried out during the treatment and not at the time of initial diagnosis, which was prior to the above-mentioned guidelines. An earlier detection of the *ATM* heterozygous variants would have probably allowed further considerations in the therapeutic management of patients and their families about cancer risk assessment.

The clinical management of patients with *ATM*-related neoplasms is not clearly defined by international guidelines, as also the risk management in unaffected family members harboring the variant. These inconsistencies, along with the non-systematic application of multi-gene testing in different conditions, lead to several uncertainties concerning the clinical significance of heterozygous *ATM* variants. The international survey on the interpretation of germline mutant alleles by ENIGMA consortium states that “the utility of a gene needs to be continuously reconsidered as more data become available, and this can only be done by analyzing results from large cohorts of individuals who have been tested” [34].

These uncertainties have been highlighted at the first international workshop of the *ATM* and cancer risk group, stating that *ATM* pathogenic variant carriers may benefit from tailored, effective cancer risk assessment and management. This may be possible by referring patients with new diagnoses and their family members to genetic counseling, expanding NGS use to better correlate the presence of such variants with clinical and biological tumoral features and to identify carriers also without a severe personal or family history and inform about therapeutic strategies in affected and carrier individuals [35].

One option to take into consideration from a prevention perspective is RRBSO. NCCN guidelines state that there is not enough evidence to recommend RRBSO based on a pathogenic germline *ATM* variant alone, but considering the uncertain clinical benefits of OC screening, one might propose RRBSO at the age of 45–50 not only in the case of positive family history (first-degree or second-degree relatives) [15, 36].

ESMO clinical guidelines [5] affirm that for women carrying *PALB2* P/LP variants the risk of ovarian cancer is 3–5% and that for *ATM* is likely 5%. At this level of risk premenopausal RRBSO is not routinely recommended. In post- menopausal women with *PALB2* P/LP variants, RRBSO can be considered. It also proposes that Polygenic Risk Score (PRS) would be a useful tool in the future particularly in individuals with *ATM* P/LP variants to capture the risk associated with Single Nucleotide Polymorphisms (SNPs) and is likely to become increasingly important as individuals without a strong family history of cancer undergo genetic testing.

Regardless of family history, Liu et al. provide a clinical guide for hereditary predisposition genes and possible role of RRBSO [37]. In this guide, they reviewed the available literature on the risk of hereditary OC for moderate-penetrance genes and they established a threshold for considering prophylactic surgery, when the benefit of RRBSO outweighs the risk of surgical morbidity and surgical menopause. This threshold range is set on 3–4% individual risk inferred for mutation status alone. Individuals with genetic variants conferring a cumulative lifetime risk for OC above this threshold, benefit from RRBSO. For *ATM* variants the cumulative lifetime risk is estimated to be up to 3–4% and it may increase in case of a positive family history [36]. This highlights the relevance of considering RRBSO as an option at least in some of these cases. To validate this option, recently an English study by T. Mukhtar et al. analyzed retrospectively and prospectively the associations between cancers and coding variants in *ATM* using whole-exome sequence data from UK Biobank (348 488 participants) and showed that the combined Relative Risk (RR) for protein-truncating variants (PTVs) in *ATM* and ovarian cancer was 3.2 [38]. Additionally, it has been demonstrated that PRS might have relevance in determining specific cancer susceptibility even in individuals with monogenic cancer susceptibility conditions [39], and this might help define subclasses of carriers who might definitely benefit from RRBSO.

The cases reported in the present work highlight the importance of periodically proposing novel genetic counseling assessment in individuals who have personal OC history and who had previous non-conclusive results. However, the degree of uncertainty is still marked. Moderate-penetrance genes might present with atypical, less informative familial and personal history as opposed to classical high-penetrance HBOC, and in many cases clinical information might be incomplete or misleading. In case B, this can be appreciated in the late onset of the ovarian neoplasm in the index patient. In the general population, the median age onset for ovarian cancer in western populations is during the 7th decade [40]. While for high-penetrance HBOC genes the age of onset has been estimated to be 10 years earlier than the general population [41], with a significant number of very early onset cases [40], information concerning moderate-penetrance genes (including *ATM*) is less solid. Individual reports usually focus on early-onset cases [42], and, while at least for other tumor types an earlier age of onset has been ascertained [30], some authors propose for *ATM* (and other moderate penetrance genes) an association mainly with late-onset ovarian cancer [40]. In Case A, the onset was with a mucinous tumor, with apparent progression to low-grade serous carcinoma and, ultimately, to HGSOC. With the available data, it is difficult to assess whether this is an actual disease progression, a series of independent events, or the result of pathological misinterpretation. Typically, HBOC displays a marked association with HGSOC as opposed to other histotypes, and this has been especially ascertained for *BRCA1/2* [43]. However, for *ATM* (and moderate-penetrance genes in general), specific association are less definite, as large studies often do not differentiate between epithelial ovarian cancer types [30], while information on specific subtypes is mainly based on isolated reports [42]. Ultimately, the relevance of the variant identified in Case A might be significant to the HGSOG diagnosis, but no further assumption can be made on the past history of the patient.

The evolution of international guidelines and clinical practice might allow novel molecular diagnoses, with significant implications for affected individuals and their families. Currently, only RRBSO remains the only effective strategy to lower the risk of developing OC. Prevention strategies for OC in *ATM* variant carriers are not defined. We suggest that additional factors—such as family history, the age at which cancer was diagnosed in relatives, and other hormonal risk factors—can be taken into account when evaluating the appropriateness of RRBSO in ATM variant carriers within a shared decision-making process.

## Materials and methods

Variant classification was carried out according to the ACMG/AMP recommendations [17] based on the latest ClinGen HBOP VCEP *ATM* specifications [19]. The identified variants were reported in accordance with the Human Genome Variation Society (HGVS) nomenclature guidelines (https://varnomen.hgvs.org/ last accessed on 26 June 2023).

### Case A

Genomic DNA was extracted from peripheral leukocytes from the proband. The targeted NGS analysis was performed on an Ion Personal Genome Machine (Ion PGM™) platform (Thermo Fisher Scientific, Carlsbad, CA, USA), with an amplicon-based Ion Ampliseq Custom Panel (Thermo Fisher Scientific, Carlsbad, CA, USA). The Torrent suite tools were used for analysis (Version 5.10, Thermo Fisher Scientific, Carlsbad, CA, USA).

### Case B

Genomic DNA was extracted from peripheral leukocytes from the proband. The targeted NGS analysis was performed on a NextSeq2000 System platform (Illumina, San Diego, California, USA), with the capture-based TruSight Hereditary cancer Panel (Illumina, San Diego, California, USA) and the NExtera DNA Flex LPK Enrichment (Illumina, San Diego, California, USA). The Dragen Enrichment_Settings Software (Illumina, San Diego, California, USA v.3.8.4) was used for analysis. Variant validation and segregation were performed by capillary electrophoresis Sanger sequencing.

## Conclusions

Data and guidelines about management of moderate penetrance genes are lacking. The expanding use of NGS allows clinicians to improve therapeutic strategies for hereditary OC, and to identify unaffected carriers that benefit from early genetic counselling to be proactively managed as opposed to regular screening, which may not always be successful. Further research is needed to better define the prognostic and therapeutic role of *ATM* P/LP variants and the possibility of resorting to RRBSO based on increasingly updated data.

## Data Availability

Sequence data cannot be shared openly, to protect study participant privacy.
